# Cardiorespiratory performance of coronary artery disease patients on land *versus* underwater treadmill tests: a comparative study

**DOI:** 10.6061/clinics/2017(11)04

**Published:** 2017-11

**Authors:** Mauricio Koprowski Garcia, Limanara Rizzo, Paulo Yazbek-Júnior, Daniela Yutiyama, Fabiola Jomar da Silva, Denise Matheus, Luiz Eduardo Mastrocolla, Eduardo Massad

**Affiliations:** I Instituto de Medicina e Reabilitacao, Hospital das Clinicas (HCFMUSP), Faculdade de Medicina, Universidade de Sao Paulo, Sao Paulo, SP, BR; II Departamento de Medicina Legal e Etica Medica, Faculdade de Medicina (FMUSP), Universidade de Sao Paulo, Sao Paulo, SP, BR; III Instituto de Cardiologia Dante Pazzanase, Sao Paulo, SP, BR

**Keywords:** Cardiopulmonary Stress Test, Immersion, Individual Capability, Exercise

## Abstract

**OBJECTIVE:**

To compare responses to a cardiopulmonary exercise test on land *versus* on an underwater treadmill, to assess the cardiorespiratory performance of coronary artery disease patients while immersed in warm water and to compare with the performance of healthy individuals.

**METHODS:**

The sample population consisted of 40 subjects, which included 20 coronary artery disease patients aged 63.7±8.89 years old, functional class I and II, according to the New York Hearth Association, and 20 healthy subjects aged 64.7±7.09 years old. The statistical significances were calculated through an ANOVA test with a (1 - β) power of 0.861. ClinicalTrials.gov: NCT00989248 (22).

**RESULTS:**

Significant differences were uncovered in coronary artery disease group regarding the variables heart beats (HB), (*p*>0.01), oxygen consumption (VO_2_), (*p*>0.01) and carbon dioxide production (VCO_2_) (*p*<0.01). Also, for the same group, in relation to the environment, water *versus* on land for HB, VO_2_, VCO_2_ and oxygen for each heart beat (VO_2_/HB) all of than (*p*<0.01). The stages for data collected featured the subject’s performance throughout the experiment, and within the given context, variables rating of perceived exertion (RPE), HB, VO_2_, VCO_2_ and VO_2_/HB (*p*<0.01) showed significant interactions between test stages and environment. Additionally, there was a significant interaction between the etiology and the test stages for the variables HB, VO_2_ and VCO_2_ (*p*<0.01). Electrocardiographic changes compatible with myocardial ischemia or arrhythmia were not observed. The subjects exhibited lower scores on Borg’s perceived exertion scale in the water than at every one of the test stages on land (*p*<0.01).

**CONCLUSION:**

This study show that a cardiopulmonary exercise test can be safely conducted in subjects in immersion and that the procedures, resources and equipment used yielded replicable and reliable data. Significant differences observed in water *versus* on land allow us to conclude that coronary artery disease patients are able to do physical exercise in water and that the physiological effects of immersion do not present any risk for such patients, as exercise was well tolerated by all subjects.

## INTRODUCTION

Physical activity in a warm water pool is traditionally used in rehabilitation facilities with patients who have different pathologies. As exercise in water can be reasonably easy to perform, it may enhance mobility, strength and physical conditioning 1. Cardiopulmonary exercise tests (CPX) conducted on land are well known. When conducted in water, however, they require additional care as well as suitable equipment and resources to comply with the provisions set forth by the “clinician’s guide to CPX in adults: as cientific statement from the American Heart Association, 2010” 2.

During immersion at the level of the manubrium, there is compression on surface veins caused by hydrostatic pressure. Blood flow in lower limbs is reversed from downward to upward through the unidirectional valves, first to the thighs and then upward to the abdomen and finally to the chest and to the heart. During immersion up to the iliac crest, changes in volume are not significant, but central venous pressure begins to increase during immersion at the level of the manubrium and eventually gets higher during total immersion 3. Arborelius et al., 1972 4, showed that during immersion at the neck level, an increase in central blood volume of 700 ml is observed, which represents a 60% increase in the central volume. One-fourth of the volume, that is 180-240 ml, is oriented to the heart, thus causing the four heart chambers to dilate 5-7. The heart volume increases by 27-30% during immersion at neck level 8. The heart, however, is not a static receptacle and its physiological response to increased volume is a rise in the force of contraction the greater the myocardial distension, the greater (more efficient) the muscle contraction, according to the Frank-Starling law 9. Most cardiovascular changes depend on water temperature and level of immersion. A progressive increase in cardiac output was observed at higher temperatures, which comprised an increase by 30% at 33°C to an increase by 121% at 39°C 10. During immersion at the neck level, systemic vascular resistance decreases by 30% as a result of reduced sympathetic vasoconstriction, and it remains low for a few hours following the first hour of immersion. This effect is also temperature dependent, as the temperature increases, the magnitude of the reduction becomes greater 3,5.

Lung blood flow also increases as a result of increased central volume and blood pressure. Average lung artery pressure ranges between 5 mmHg on land and 22 mmHg during immersion at the neck level. Most of the lung blood volume is directed to the larger vessels of the lung vascular bed 6. Immersion is also associated with renal responses such as increased diuresis, decreased plasma concentration, natriuresis, kaliuresis and suppression of arginine vasopressin, plasma rennin and aldosterone, with consequent augmentation of renal free water excretion 3,9.

Several studies have assessed and validated the use of aquatic therapy for cardiovascular rehabilitation following heart failure and ischemic heart disease. Common sense clinical opinion is that immersion in warm water and controlled exercises are well tolerated by patients diagnosed with coronary artery disease (CAD), even when they experience the physiological effects discussed herein. The assumption is that there are no major differences between a CPX conducted in water and its counterpart on land. Additionally, the required equipment and resources are supposed to be similar, provided that there is compliance with the required test preparation methodology for the CPX conducted in water.

Thus, the aim of this study was to compare responses to a cardiopulmonary exercise test (CPX) on land *versus* on an underwater treadmill, to assess the cardiorespiratory performance of coronary artery disease (CAD) patients while immersed in warm water and to compare with the performance of healthy (H) individuals.

## METHODS

The sampling population of this study comprised 40 subjects, 20 of whom were healthy controls and 20 of whom were patients enrolled in the physical conditioning program of the Institute of Medicine and Rehabilitation, Clinics Hospital, School of Medicine, University of São Paulo. The patient group had a medical history of one or more of the following conditions: acute coronary syndrome, coronary angioplasty, revascularization surgery or CAD. Patients were between 55 and 80 years old, and they were first examined by a heart specialist who assessed their clinical history and validated their medical conditions. Any patient who did not comply with the test schedule or those who had peripheral artery disease, diabetes, chronic lung disease, hypertension (blood pressure >160/90), or significant or unstable cardiovascular morbidity conditionswhile taking medication were excluded from the study (Table 1). ClinicalTrials.gov: NCT00989248.

### Test Design

Both groups underwent two CPX tests and were always monitored by the same cardiology specialist throughout the testing and that did the data interpretation. All of the participants received clear explanations about the test methodology and granted their consent, after which they were requested to sign an informed consent.

Clinical data were then collected, and body mass and height were measured. Within an interval of 3-7 days after the land CPX was conducted, the second CPX was conducted in a warm indoor pool with 48 m^3^ of water volume and depths ranging between 1.10 and 2.10 m. The nursing staff prepared the subjects for both tests by first cleaning the skin areas where electrodes were applied, connecting the cables, putting the facemasks on the subjects and finally checking their systolic and diastolic blood pressure (SDBP). The gas analyzer was calibrate prior to every run of the test.

On land, the environment temperature was kept stable at 20±2°C and the relative air humidity was maintained at 50-70% 2. In the pool, the water temperature was maintained at 33-34°C and the relative air humidity between 60 to 80%. Subject test preparation for the water test followed the same procedures as for the on land test. To improve the conduction of electrical impulses without causing any signal interference, however, the electrocardiogram electrodes received additional protection by applying a 3M occlusive bandage on the top of each of the cable terminals. The subject’s adaptation to the water was check by a pool specific practical test in order to identify and exclude from the study those individuals who showed difficult adaption to or fear of the water. A brief simulation allowed subjects to become acquainted with the environment and the equipment, thus lowering their level of anxiety.

Due to a lack of interfaces connected directly the under-water treadmill to a computer, the Bruce Protocol 20 was used to be the most suitable. Perceived levels of exertion and/or dyspnea were assessed as per the Borg rating of perceived exertion 21.

A set comprising a facemask covering the nose and mouth, a cap and a flow sensor was used to conduct the air exhaled by subjects to the gas analyzer. An model Metalyzer II (Cortex CPET) measured respiratory and metabolic variables. As this device was connected to the Ergo-Elite software on the computer, it supplied the following data: exhaled gas (average min), oxygen consumption (VO_2_), carbon dioxide production (VCO_2_), oxygen equivalents (VE/VO_2_) and carbonic dioxide equivalents (VE/VCO_2_) as well as the respiratory exchange rate (RER - VCO_2_/VO_2_).

Meditrace electrodes connected to a special cable with five thoracic derivations sent electrical signals to the digital Micromed ECG, thus allowing for continuous ECG monitoring by the researcher. A Model MF621 sphygmomanometer was placed on each subject’s right arm to check SDBP. During the water test, due to the unfavorable access conditions, SDBP was checked at the beginning of the test and two minutes after the test was completed.

**Figure 1:**
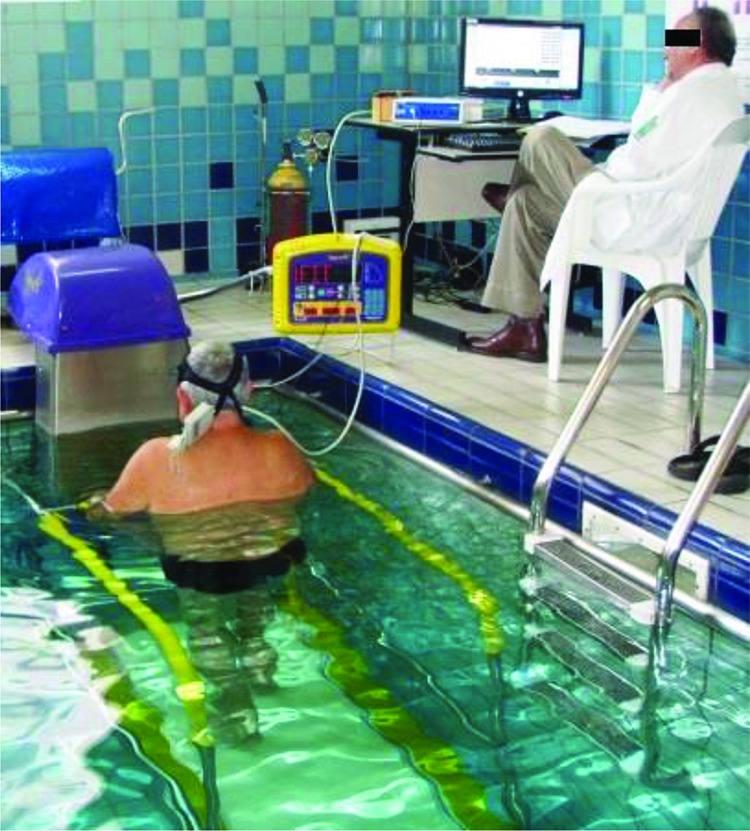
Test conducted on an underwater treadmill under supervision of a cardiologist.

For the land test, the KT-ATL Millenium treadmill by Inbramed/ Inbrasorpt® was used and for the water test, the Aquafit Hi-Tec® by Sahinco. The latter, made in stainless steel and featuring an electric-hydraulic unit, prevented accidental electric shock. Produced speeds ranging from 2 up to 10 km/h at 0-13% inclines, same inclination as the land treadmill, through electronic programming without a computer interface (Figure 1).

The test started at stage 1 of the Bruce Protocol, with 3 minutes of activity at a 10% incline and a speed of 2.7 km/h, and was completed when the Respiratory Quotient (RQ) reached ≥1.1. After the test was completed, subjects kept walking on the treadmill for 2 minutes at a speed of 2.7 km per hour and 0% incline while still being monitored. The test was interrupted whenever any of the criteria set by the Brazilian Cardiology Society/American Heart Association were not complied with 12.

**Figure 2:**
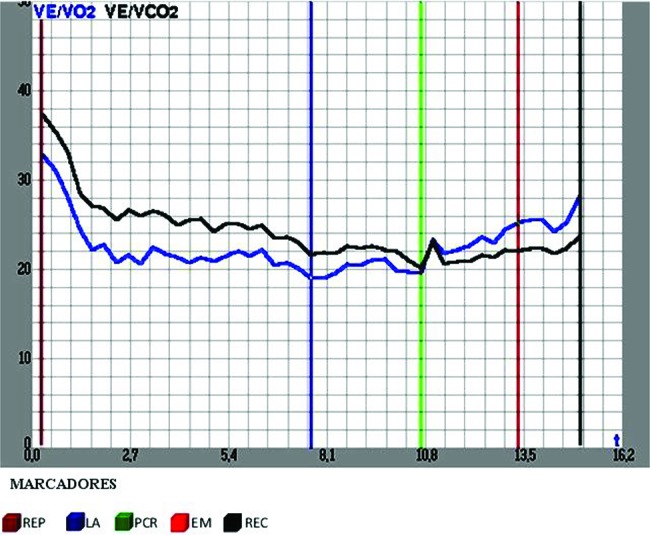
Data collected at five moments, both on land and in water.

Data were collected at 5 relevant test stages or cardiorespiratory levels: One, rest (REP), accelerated ventilation, heart beat and oxygen consumption were observed to indicate whether subjects were anxious or experiencing metabolic disorders. Two, the anaerobic threshold (AT) featured an exertion level at which CO_2_ rates increased and metabolism changed from aerobic to anaerobic. This variable shows individual capacity for exercise and the physiologic responses. Three, respiratory compensation point (RCP) showed the aerobic and anaerobic rates. This is the point when the individual is no longer able to endure lengthy exercise as his or her capacity to remove lactate decreases and the anaerobic system takes over the aerobic system. Four, maximum effort (ME), a marker of cardiopulmonary system limits. Five, recovery (REC) showed heart rate recovery rates, any cardiac stress and the rate of metabolic acidosis after exertion that leads to faster and deeper breathing, as the body tries to eliminate excessive acid by reducing the amount of carbon dioxide (Figure 2).

For safety purposes, the experimental environment featured an emergency exit, a team of experts, continuous supervision and monitoring, and an emergency cart equipped with a defibrillator and emergency drugs.

Experiment profile for the five stages, environment and study group in each variable (Table 2).

### Statistical Analysis

The stage intra-group dependent variables for both on land and in-water tests are RPE, HB, VO_2_, VCO_2_, VO_2_/HB, VE/VO_2_, VE/VCO_2_, and VCO_2_/VO_2_. The environment intra-group dependent variable stands for both the on land and in water experiment, and the etiology inter-group independent variables are CAD patients and H individuals. As this was not a treatment in itself, the analogy with a control case experiment was chosen.

A variance analysis of repeated measurements (rANOVA) was conducted for factors and for every one of the dependent variables. Time gaps between the stage variables were considered equivalent and were not compromised by the intra-group error term. As non-homogeneous variances were expected throughout the test stages, Greenhouse-Geisser or Huyn-Feldt corrections 23 were conducted according to the sphericity deviation score, as assessed by the Mauchly test 24.

As there is no canonical methodology for identifying significant differences in this type of variance analysis, it was decided to use t-tests for every one of the possible combinations between the three factors under investigation and Holm-Bonferroni corrections for multiple tests. In both cases, the significance level was set at 5%.

The sampling population profile is shown by tables with descriptive statistics (means and standard deviations) and is illustrated by graphs of the mean profiles and boxplots for every factor. ANOVA statistical power was calculated at (1 - β)=0.861, thus totaling a minimum of 20 participants per group.

### Ethical Aspects

This study was approve by the ethical review board of school of Medicine – University of Sao Paulo, protocol number 0532/08. All subjects agreed to volunteer for the study and signed an informed consent form.

## RESULTS

The analysis showed major effects for CAD group *versus* Health one for the variables HB, VO_2_ and VCO_2_, all of than with (*p*>0.01), no significant effect for the other variables. The major effect for environment, land *versus* water, showed significance for variables HB, VO_2_, VCO_2_ and VO_2_/HB, all of than with (*p*>0.01) (Table 3).

In addition to the major effects described above, it is worth noting that the interactions between factors, particularly the interactions with stage, indicate the subject’s performance throughout the experiment. In this context, the variables RPE (*p*>0.01), HB (*p*=0.02), VO_2_ (*p*>0.01), VCO_2_ (*p*>0.01), and VO_2_/HB (*p*>0.01), suggested significant interactions between stage and environment. In other words, the on-land mean profiles were different from the in water profiles throughout the experiment. Moreover, there was a significant interaction between etiology and stage for variables HB (*p*>0.01), VO_2_ (*p*>0.01), and VCO_2_ (*p*>0.01). CAD patients and H individuals showed different performances throughout the experiment for these variables. There were no significant interactions regarding the others variables. Furthermore, there was no significant interaction between etiology and environment; these factors can be interpreted independently, which there for e justifies the method chosen for the variance analysis and data interpretation (Table 4).

**Figure 3:**
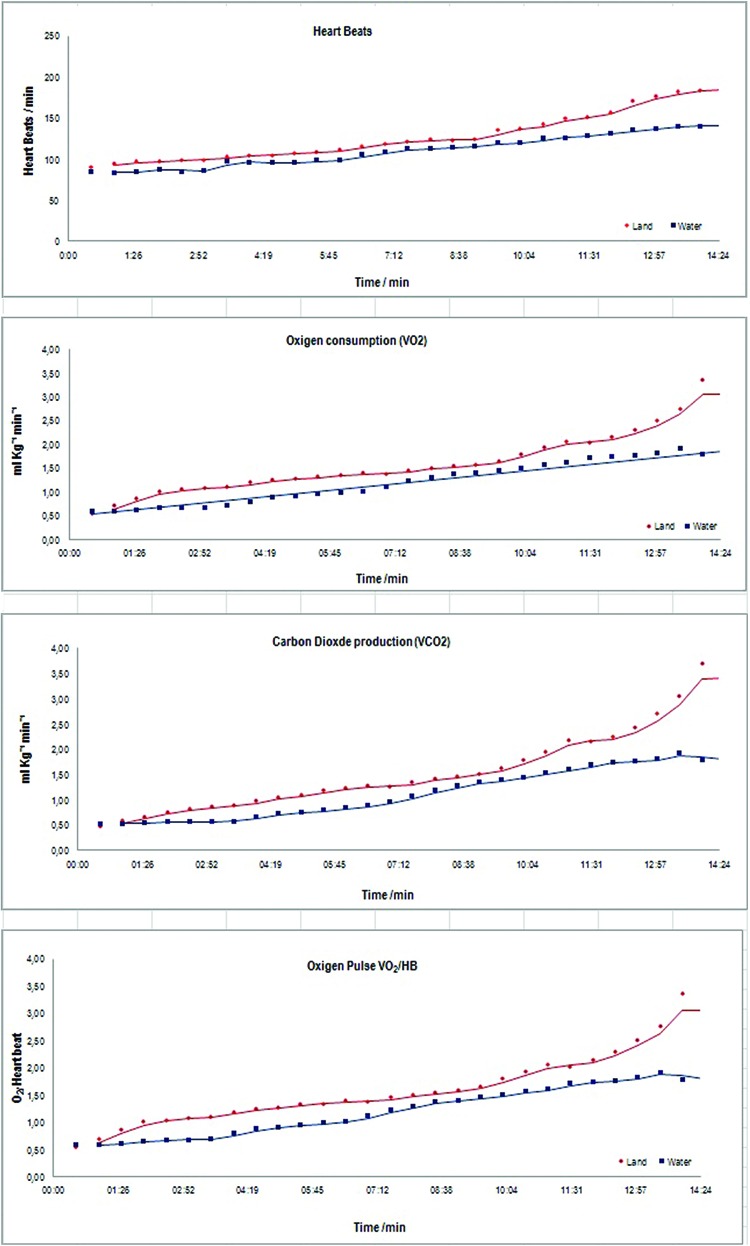
Major effects CAD and Health groups for land *versus* water, profile for test time measured in minutes and seconds.

Major effects CAD and Health groups for land *versus* water, profile for test time measured in minutes and seconds (Figure 3).

The Bruce Protocol determines changes in the treadmill incline and speed every 3 minutes, thus resulting in changes in the variables under investigation in the 5 specific set-points of data collection. Hemodynamic changes and cardiovascular overload caused by immersion might lead to a shorter exertion period in the in water test. However, the opposite was observed for both CAD patients and H individuals; the activity time in water was longer than on land (*p*<0.001). ECG changes compatible with myocardial ischemia or arrhythmia were not observed, and the SDBP did not vary significantly.

## DISCUSSION

In this study, we tested the hypothesis that immersion in warm water and the physiological effects associated with exercise during immersion would not be risky for patients with CAD. This study further aimed to investigate and produce reliable and replicable data about cardiorespiratory responses during a CPX conducted in water compared with the test conducted on land. The “Clinician's Guide to Cardiopulmonary Exercise Testing in Adults” from the American Heart Association 2 clearly states that CPX conducted on land is an extremely safe and versatile test that provides data for evaluating a wide range of responses and levels of tolerance to physical exercise. This set of data is invaluable for the diagnosis and prognosis of patients with cardiovascular diseases.

Conducting a CPX in water, however, is not an ordinary practice, as it requires specific equipment, resources and procedures to ensure accurate records and test replicability and to make this test as reliable as its counterpart on land. The quality of data depends on several factors, such as subject preparation for capturing ECG signals without any interference using a 5 derivation cable and electrodes covered with occlusive bandages.

The major contribution of this study is that it demonstrated that exercise in warm water is well tolerated by patients with CAD and that conducting a CPX in water is feasible and safe. In addition, our results showed that walking or jogging on an underwater treadmill with water at chest level requires less cardiopulmonary exertion than doing so on land. CAD patients showed as high a level of tolerance during the in-water test as the control group subjects, and no adverse events were observed.

Issues associated with cardiac preload and post load during immersion were observed as a result of blood displacement, especially from the lower limbs upward to the thoracic cave, thus increasing the volume of blood filling the four heart chambers. Blood volume increases by 27-30% when subjects are immersed to neck level 7. The heart, however, is not a static receptacle and the physiological cardiac response to increased blood volume causes the cardiac muscle to contract more forcefully as the healthy myocardium stretches. According to the Frank and Starling Law 8, contraction efficiency will improve. Gabrielsen et al., 2000 and 1993 13 showed that both cardiac output and stroke volume increase during immersion in water at 30°C 14.

Immersion to the xiphoid process reduces the load on joints and bones by at least 60% 15. The effect of up thrust or buoyancy is an upward vertical force exerted by a fluid and the magnitude of that force is proportional to the weight of the displaced water volume by the object; buoyancy can thus facilitate exercise in water as the force of gravity is reduced.

The resistance of the water, however, which is 800 times denser than air, should theoretically require more effort to walk or jog in on an underwater treadmill. Nevertheless, this study shows quite the opposite. As subjects walked or jogged on the underwater treadmill, they did not fight the resistance of the water, and up thrust became a facilitating force as it demanded less exertion of the anti-gravitational muscles to support their body weight. Consequently, the perceived exertion scale Borg RPE showed statistically significant differences between in water and on land tests (*p*<0.01) for both groups in every stage. Christie et al., 1990 11, demonstrated that the heart rate in healthy subjects has similar responses to exercise on land and in water until VO_2_ reaches 60% of VO_2peak_. Our findings demonstrated that the heart rate exhibits significant effects and interactions between etiology and stage, (*p*<0.01), stage and environment (*p*<0.01). At all of the stages, the HB rate was higher on land (*p*=0.02) than in water (*p*<0.01) for both groups and healthy subjects had a higher HB rate than CAD patients in both environments. This can be explained by the use of medication - CAD group.

VO_2_ seems to be affected by several factors, but the magnitude of oxygen consumption is a much more reliable indicator of cardiorespiratory functional capability 12. In 1989, Gleimand Nicholas 16 demonstrated that oxygen consumption during a walking or jogging exercise at a speed of 53 m/min was three times greater in water than on land. Therefore, in water, only one-half to one-third of the speed is required to reach the same metabolic intensity rate as on land performance. Because subjects did not run against the resistance of water in this study, this feature might have hindered the collection of similar findings to those in Gleimand Nicholas’ study. Nevertheless, our findings show the significant effects and interactions between etiology and stage, (p<0.01) and stage and environment (*p*<0.01). VO_2_ on land was higher than in water at all the stages for both CAD and Healthy subjects and both showed lower VO_2_ in water than on land (*p*<0.01). Craig and Dvorak, 1969 14, showed that at rest, VO_2_ was similar for healthy subjects on land and in water at 34°C. This finding, however, could not be replicated in the current study, which suggests that the resistance of water or the temperature (34°C) affected oxygen consumption. CO_2_ production shows a causal relationship with O_2_ and a linear increase.

According to Yazbek et al., 1998 12, the O_2_ pulse VO_2_/HB indicates the amount of O_2_ transported during every heart beat. Lower VE performance may be detected in an increasing exertion test by observing the VO_2_/HB, and it represents an indirect index of O_2_ transported by the cardiopulmonary system. Significant effects and interactions were observed for stage and environment (*p*<0.01), but not for etiology and stage (*p*=0.75). According to Hall et al., 1990 17, a reduced-gravity environment requires less activity of the anti-gravity muscles, which might explain the significant difference observed in the VO_2_/HB, which was lower in water than on land for both groups (*p*<0.01). However, it does not explain why CAD patients showed lower O_2_ pulse (*p*<0.01) in water and on land than healthy individuals.

The relationships of VE/VO_2_ and VE/VCO_2_ with VE at body temperature pressure saturated (BTPS) conditions and VO_2_ and VCO_2_ at standard air temperature and air pressure dry (STPD) conditions show how many liters of air per minute are necessary and must be ventilated to consume 100 ml of O_2_ and to produce CO_2_.

This study has assessed that even during increasing exertion and under the physiological effects of immersion, both CAD patients and H individuals did not show significant changes in VE, which remained appropriate to the relative demand of O_2_ at each of the stages. In a previous study, Craig and Ware, 1967 18, and Hall et al., 1990 19, showed that for healthy individuals, VE decreases during immersion. These findings, however, were not observed in the current study. Our findings corroborate those of Asa C. et al. 20. Ventilatory efficiency during exercise, both on land and in water on a treadmill, was according to the demand of O_2_.

Respiratory quotient or QR reflects the quotient between VCO_2_ and VO_2_. During increased exertion, which was the case in this study, metabolism increasingly uses carbohydrates as a source of energy, reaching ≥1.1 at maximum exertion. In both tests (in water and on land), subjects reached a QR>1, so there was no significant effect between stage and environment and etiology and environment.

As hemodynamic responses to exercise vary depending on cardiac output and peripheral resistance, increased systolic blood pressure was expected as a result of higher cardiac output in both environments, mainly during immersion. Diastolic blood pressure, in contrast, was expected to remain stable or even to mildly decrease with exercise. However, no significant SDBP response was observed. Studies concerning the SDBP responses of healthy individuals during rest at thermo neutral temperature immersion are conflicting and scarce in the literature Gabrielsen et al., 1993 13.

No significant arrhythmia or ventricular ectopic activity and/or instances of ST-segment depression were observed by the cardiologist in the ECG recordings performance evaluation curve.

### Study limitations

The use of an underwater treadmill without any computer interface prevented this study from automatically controlling the treadmill speed and incline. This might have affected data collection for the in water test, however, the methodology used for both tests was identical and the subjects had to walk or jog in compliance with the Bruce Protocol 10.

The order of the test was not randomized because a CPX in water is not well known and, for safety, we prefer to start with a CPX on land, secure and widely used procedure.

This study can be presents a small study population although we performed more 144 tests, but many had to be discarded because of the difficulties in collecting data, primarily in the immersion test. That is why we used the statistical power was calculated at (1 - β)=0.861, thus totaling a minimum of 20 participants per group.

This study show that a CPX can be safely conducted in subjects in immersion and that the procedures, resources and equipment used yielded replicable and reliable data. Significant differences observed in water *versus* on land allow us to conclude that CAD patients are able to do physical exercise in water and that the physiological effects of immersion do not present any risk for such patients, as exercise was well tolerated by all subjects. We can also conclude that CPX conducted in water may be used by cardiologists to safely diagnose and estimate the exertion capability of CAD patients to perform dynamic exercise in a pool. Further investigation is necessary to provide a clearer understanding of the physiological effects of immersion associated with higher risk subjects.

A cross-sectional study where the effects of medium and long term are not known, but this was not the objective of the study.

## AUTHOR CONTRIBUTIONS

Garcia MK was responsible for the study conception and design, analysis and interpretation of data and manuscript writing. Rizzo L was responsible for the coordination of the research, conduction of the experiments and manuscript writing. Yazbek-Júnior P was responsible for the conduction of the experiments with cardiopulmonary stress exertion analysis and interpretation of data. Yutiyama D, Silva F.J and Matheus D were responsible for the conduction of the experiments. Mastrocolla LE was responsible for the analysis and interpretation of the findings. Massad E was responsible for the study conception, design, analysis and interpretation of the findings.

## Figures and Tables

**Table 1:** Baseline characteristics of subjects.

	CAD	SD	Healthy	SD
N	20		20	
Males	20		20	
Age (years)	63.7	±8.89	64.7	±7.09
Weight (Kg)	73.7	±10.6	73.6	±18.8
Height (cm)	168	±5.95	170.6	±11.7
Body Mass Index - BMI (kg/m^?^)	22.83		23.19	
Patients Under Medication				
Antihypertensive	18			
Diuretic	6			
Hypercholesterolemia	9			
Functional class I VO_2_ max >20ml/kg^-1^ min^-1^	8			
Functional class I VO_2_ (AT)>14ml/kg^-1^ min^-1^	8			
Functional class II VO_2_ max from 16 to 20mL/kg-1 min^-1^	12			
Functional class II VO_2_ (AT) from 11 to 14mL/kg^-1^ min^-1^	12			
Base Etiology - Revascularization/Stent	12/8			

Coronary Artery Disease (CAD); Standard Deviation (SD); Number of participants (n); Functional Class as per the New York Heart Association (NYHA). Maximum rate of oxygen consumption (VO_2_ max); Oxygen volume (VO_2_); Anaerobic Threshold (AT).

**Table 2:** Experiment profile for the five stages, by environment and study group in each variable.

Stage	Environment	Etiology	RPE		HB		VO_2_		VCO_2_		VO_2_/HB		VE/VO_2_		VE/VCO_2_		VCO_2_/VO_2_	
			µ	SD	µ	SD	µ	SD	µ	SD	µ	SD	µ	SD	µ	SD	µ	SD
REP	Pool	H	7.15	0.49	85.90	12.56	0.64	0.16	0.54	0.14	7.66	2.23	30.67	6.97	36.03	6.28	0.85	0.08
		DAC	7.40	0.82	81.45	13.00	0.57	0.17	0.49	0.12	7.00	1.92	31.82	9.37	36.09	6.39	0.87	0.14
	Land	H	7.10	0.45	88.60	11.49	0.59	0.17	0.53	0.19	6.66	1.82	31.18	10.86	35.52	6.31	0.90	0.16
		DAC	7.30	0.73	81.30	15.62	0.56	0.19	0.46	0.14	6.89	2.49	30.88	10.21	35.89	7.97	0.84	0.12
AT	Pool	H	9.95	1.05	103.95	13.80	1.26	0.37	1.02	0.31	12.19	3.16	25.00	3.52	30.81	4.03	0.87	0.23
		DAC	10.30	1.22	90.00	15.45	0.97	0.33	0.78	0.24	10.80	2.88	26.68	4.05	32.92	5.05	0.81	0.05
	Land	H	9.25	1.37	109.10	9.79	1.53	0.40	1.28	0.35	13.97	3.75	24.30	3.21	29.23	3.94	0.84	0.11
		DAC	9.85	1.60	9.45	17.82	1.22	0.23	0.99	0.20	13.12	3.21	25.92	5.90	31.77	5.85	0.81	0.10
RCP	Pool	H	12.40	1.50	122.90	10.74	1.69	0.53	1.58	0.49	13.65	4.00	27.95	4.17	29.66	3.84	1.02	0.39
		DAC	12.10	1.07	102.40	17.14	1.20	0.35	1.13	0.33	12.17	2.59	30.29	5.20	32.12	4.70	0.94	0.10
	Land	H	11.25	1.62	127.65	8.63	1.83	0.50	1.71	0.40	14.40	4.06	27.77	4.41	29.30	4.10	0.95	0.11
		DAC	12.15	1.46	108.30	18.48	1.44	0.27	1.33	0.27	13.62	3.03	28.69	7.22	30.93	5.68	0.93	0.13
ME	Pool	H	14.85	1.31	139.35	13.29	1.96	0.66	2.17	0.71	14.52	3.92	34.69	5.99	31.89	4.56	1.09	0.15
		DAC	14.85	1.35	118.60	20.63	1.51	0.33	1.60	0.38	12.92	2.86	35.27	6.31	33.30	5.02	1.06	0.12
	Land	H	15.00	1.62	149.65	12.77	2.35	0.85	2.61	0.94	15.90	5.31	35.63	6.20	31.77	5.62	1.13	0.15
		DAC	15.20	1.01	126.50	17.82	1.72	0.36	1.78	0.31	15.33	4.98	34.91	7.92	33.77	5.74	1.04	0.12
R	Pool	H	15.05	2.72	99.85	12.76	0.85	0.26	1.15	0.35	8.95	3.17	46.41	10.30	33.10	4.93	1.41	0.30
		DAC	15.60	0.88	89.30	14.12	0.68	0.21	0.93	0.22	7.13	2.29	50.17	11.41	35.85	6.66	1.41	0.24
	Land	H	16.10	1.17	109.15	11.39	1.13	0.41	1.51	0.53	10.32	3.48	45.12	11.49	33.45	5.49	1.37	0.42
		DAC	16.45	0.83	89.95	22.62	0.91	0.24	1.11	0.29	10.39	2.67	44.05	11.26	35.20	5.82	1.25	0.22

Stages: Rest (REP); Aerobic Threshold (AT); Respiratory compensation point (RCP); Maximum effort (ME); Recovery (R); Borg Rating of Perceived Exertion (RPE); Heart Beat (HB).

Variables: Oxygen Consumption (VO_2_); Carbon Dioxide Consumption (VCO_2_); Oxygen Pulse (VO_2_/HB); Oxygen Equivalents (VE/VO_2_); Carbon Dioxide Equivalents (VE/VCO_2_); Respiratory Exchange Rates (VCO_2_/VO_2_).

Mean (µ); Standard Deviation (SD); Health (H); Coronary artery disease (CAD).

**Table 3:** Major effects for every variable under investigation.

Variables	CAD *vs* Healthy		Land *vs* Water	
	F	*p*	F	*p*
RPE	2.81	0.10	0	1.00
HB	17	<0.01	13	<0.01
VO_2_	12.2	<0.01	25.6	<0.01
VCO_2_	16.5	<0.01	25.4	<0.01
VO_2_/HB	1.39	0.25	15.4	<0.01
VE/VO_2_	0.45	0.51	1.51	0.23
VE/VCO_2_	1.35	0.25	1.76	0.19
VCO_2_/VO_2_	1.91	0.17	1.17	0.29

RPE-Borg Rating of Perceived Exertion; HB-Heart Beat; VO_2_-Oxygen Consumption; VCO_2_-Carbon Dioxide Consumption; VO_2_/HB-Oxygen Pulse; VE/VO_2_-Oxygen Equivalents; VE/VCO_2_-Carbon Dioxide Equivalents; VO_2_/VO_2_-Respiratory Exchange Rates; F-Statistics; *p*-Significance.

**Table 4:** Interactions between Etiology *vs* Stage and Environment *vs* Stage.

Variables	Etiology x Stage		Environment x Stage	
	F	*p*	F	*p*
RPE	0.32	0.87	5.67	<0.01
HB	7.1	<0.01	3.01	0.02
VO_2_	6.4	<0.01	10.1	<0.01
VCO_2_	8.17	<0.01	8.43	<0.01
VO_2_/HR	0.48	0.75	6.79	<0.01
VE/VO_2_	0.24	0.30	1.72	0.15
VE/VCO_2_	1.59	0.18	0.86	0.49
VCO_2_/VO_2_	0.23	0.92	1.89	0.12

RPE-Borg Rating of Perceived Exertion; HB- Heart Beat; VO_2_-Oxygen Consumption; VCO_2_-Carbon Dioxide Consumption; VO_2_/HB-Oxygen Pulse; VE/VO_2_-Oxygen Equivalents; VE/VCO_2_-Carbon Dioxide Equivalents; VO_2_/VO_2_-Respiratory Exchange Rates; F-Statistics; *p*-Significance.
